# Effects of an 11-nm DMSA-coated iron nanoparticle on the gene expression profile of two human cell lines, THP-1 and HepG2

**DOI:** 10.1186/s12951-014-0063-3

**Published:** 2015-01-17

**Authors:** Ling Zhang, Xin Wang, Jinglu Zou, Yingxun Liu, Jinke Wang

**Affiliations:** State Key Laboratory of Bioelectronics, Southeast University, Nanjing, 210096 China; School of Biomedical Engineering, Hubei University of Science and Technology, Xianning, 437000 China

**Keywords:** Iron nanoparticle, Gene expression profile, THP-1, HepG2

## Abstract

**Background:**

Iron nanoparticles (FeNPs) have attracted increasing attention over the past two decades owing to their promising application as biomedical agents. However, to ensure safe application, their potential nanotoxicity should be carefully and thoroughly evaluated. Studies on the effects of FeNPs on cells at the transcriptomic level will be helpful for identifying any potential nanotoxicity of FeNPs and providing valuable mechanistic insights into various FeNPs-induced nanotoxicities.

**Results:**

This study investigated the effects of an 11-nm dimercaptosuccinic acid-coated magnetite nanoparticle on the gene expression profiles of two human cell lines, THP-1 and HepG2. It was found that the expression of hundreds of genes was significantly changed by a 24-h treatment with the nanoparticles at two doses, 50 μg/mL and 100 μg/mL, in the two cell types. By identifying the differentially expressed genes and annotating their functions, this study characterized the general and cell-specific effects of the nanoparticles on two cell types at the gene, biological process and pathway levels. At these doses, the overall effects of the nanoparticle on the THP-1 cells were the induction of various responses and repression of protein translation, but in the HepG2 cells, the main effects were the promotion of cell metabolism, growth and mobility. In combination with a previous study, this study also characterized the common genes, biological processes and pathways affected by the nanoparticle in two human and mouse cell lines and identified *Id3* as a nanotoxicity biomarker of the nanoparticle.

**Conclusion:**

The studied FeNPs exerted significant effects on the gene expression profiles of human cells. These effects were highly dependent on the innate biological functions of cells, i.e., the cell types. However, cells can also show some cell type-independent effects such as repression of *Id3* expression. *Id3* can be used as a nanotoxicity biomarker for iron nanoparticles.

**Electronic supplementary material:**

The online version of this article (doi:10.1186/s12951-014-0063-3) contains supplementary material, which is available to authorized users.

## Background

Iron nanoparticles (FeNPs) have attracted increasing attention over the past two decades owing to their promising applications as biomedical agents [1,2]. FeNPs have been the most intensively studied and commercialized nanomaterial in recent years. Despite their generally good biocompatibility relative to other metal nanomaterials [3], their potential nanotoxicity has been recognized [4,5]. For this reason, many studies have investigated the potential nanotoxicity of FeNPs [6]. Some important nanotoxicities of FeNPs were thus discovered, including reduction of cell viability [7,8] and induction of cellular inflammation [9,10], mitochondrial injury [8,11,12], apoptosis [8,13,14], reactive oxygen species (ROS) [8,11,15,16], autophagy [8,11], oxidative stress [14,17,18], cell motility impairment [15], and DNA damage [17,18].

In response to any intracellular and extracellular environmental changes, cells can rapidly change their transcriptomic output, i.e., gene expression profile. In this way, cells adapt to the environmental changes for their survival and function. However, excessive environmental changes can damage the normal physiological activities and biological functions of cells. Therefore, evaluation of gene expression profile changes is helpful in identifying the potential nanotoxicity of nanomaterials [16,19-21]. Identifying all the genes whose expression is affected by a nanomaterial at the cell or tissue levels can provide valuable clues for identifying any potential toxicity and the relevant molecular mechanism [16,19,20,22]. Moreover, current transcriptomic profiling techniques including GeneChip and RNA-seq allow the analysis of global gene expression [20,23,24]. Therefore, increasing numbers of studies have investigated the nanotoxicity of various nanomaterials at the transcriptome level [16,20,24]. Many previously unknown nanotoxicities of nanomaterials were thus uncovered, such as intracellular production of ROS and the resulting cell apoptosis induced by silver, silica and magnetic nanoparticles [19,20,25].

Recent transcriptomic studies have provided valuable mechanistic insights into the various nanotoxicities induced by FeNPs. For example, a transcriptomic analysis found that the transcription of many genes relevant to iron metabolism (*Trf*, *Tfrc*, *Lcn2*, *Hfe*) and osmosis (*Slc5a3*, *Slc6a12*) was significantly changed by FeNPs in mouse RAW264.7 cells [21], indicating that the iron and osmotic homeostasis of the cells was disturbed by FeNPs. The subsequent measurement of the cellular iron content revealed that the internalized FeNPs were degraded in the acidic environment of the lysosomes and thus released iron ions in the cells, which changed the iron and osmotic homeostasis of the cells. In complementary responses, the cells downregulated the expression of the *Trf*, *Tfrc*, and *Hfe* genes to prevent the transfer of extracellular Fe^2+^ into the cells, upregulated the expression of the *Lcn2* gene to promote the transfer of intracellular Fe^2+^ out of the cells, and downregulated the expression of the *Slc5a3* gene to inhibit the transfer of extracellular myo-inositol, a very important organic osmolyte, into the cells [21].

Our lab has recently evaluated the effects of a FeNP material deemed to have good biocompatibility, 11-nm magnetite (Fe_3_O_4_) FeNPs coated with dimercaptosuccinic acid (DMSA) [9], at the transcriptome level. The potential nanotoxicological effects of these FeNPs at doses of 50 and 100 μg/mL on the gene expression profiles of two mouse cell lines (RAW264.7 and Hepa1-6) were examined [10]. This study characterized the general and cell-specific biological processes affected by the FeNPs in these two cell lines by identifying the differentially expressed genes (DEGs) and annotating their functions, providing new insights into the nanotoxicity of the FeNPs. RAW264.7 cells are a blood cell line belonging to monocyte-macrophage system, whereas Hepa1-6 cells are a liver-derived hepatoma cell line. Generally, the former is mainly involved in immune activity, whereas the latter is responsible for detoxification in the living body. The blood and liver cells encounter the greatest exposure to the nanomaterials in vivo due to the use of intravenous administration and the passive targeting of nanomaterials. Therefore, the two cell lines are suitable for evaluating the nanotoxicity of FeNPs.

The benefit of using mouse cells is that the nanotoxicity observed *in vitro* can be further evaluated *in vivo* by administering the nanomaterials to mice [26]. However, the similar *in vivo* evaluation cannot be performed in humans. Therefore, a feasible strategy is to evaluate the nanotoxicity of a nanomaterial with human cells and their mouse equivalents. If the *in vitro* nanotoxicity of a nanomaterial is similar in cells of two species, its *in vivo* nanotoxicity can be evaluated in the mouse to judge its *in vivo* nanotoxicity in humans. According to this strategy, based on our recent study of the nanotoxicity of a FeNP with two mouse cells [10], this study treated two equivalent human cell lines, human monocytic THP-1 cells and hepatoma HepG2 cells, with the same FeNPs at the same doses (50 and 100 μg/mL) for the same time (24 h), and profiled the global gene expression with genechips. This study thus identified hundreds of DEGs in two cell lines. By comparing the DEGs, their annotated functions and the associated pathways, this study evaluated the general and cell-specific effects the FeNPs on two human cell lines. By comparing these results with the previously characterized effects of the same FeNPs on two mouse cell lines, this study defined the common effects of the FeNPs on human and mouse cells. This study also identified a cell-independent nanotoxicity biomarker for the FeNPs. Together, the results of this study provide new insights into the nanotoxicity of the FeNPs and the underlying molecular mechanisms.

## Results and discussion

### Characterization of FeNPs and their cellular internalization

**Figure 1:**
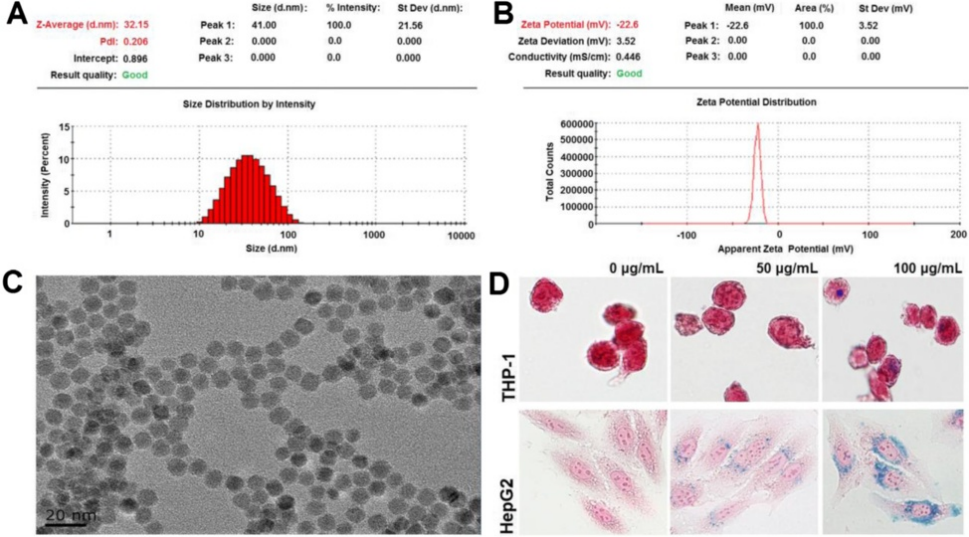
**Characterization of the FeNPs and their cellular internalization. A**: Hydrodynamic sizes of the FeNPs. **B**: Zeta potential of the FeNPs. **C**: TEM observation of FeNPs. **D**: Prussian blue staining of cells treated with FeNPs at three doses. Magnification, ×400.

The average hydrodynamic size of the FeNPs was 32 nm (Figure 1A). Zeta potential measurements showed that the FeNPs were negatively charged in water (Figure 1B). The average size of the FeNPs measured by TEM was 11 ± 1.24 nm. The FeNPs were monodisperse and of uniform size in water (Figure 1C). Prussian blue staining revealed that the FeNPs were taken up by the cells and more nanoparticles were internalized into the cells at the high dose (Figure 1D). The blue staining of the FeNPs agglomerates was clearer in the HepG2 cells than in the THP-1 cells. The reason for the different appearance is that the former is an adherent cell but the latter is a suspension cell.

### Identification of FeNP-responsive genes

**Figure 2:**
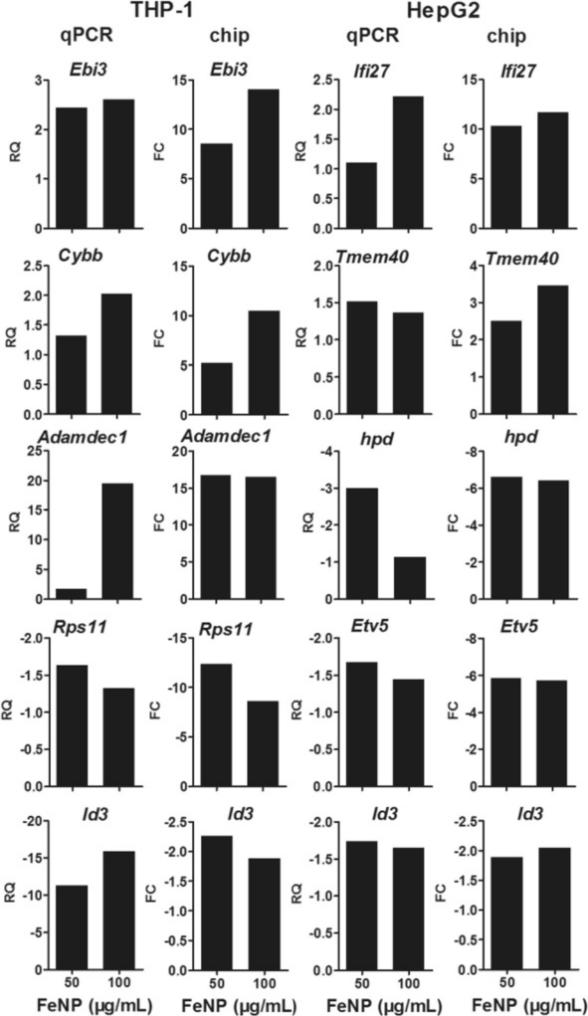
**Quantitative PCR (qPCR) detection of the transcription of genes.** The transcription of 5 genes in each type of cells was detected with qPCR. The relative quantification (RQ) of the qPCR detection was compared with the fold change (FC) of the GeneChip detection.

The GeneChip analysis identified 287 and 714 genes as DEGs (i.e., FeNP-responsive genes, FeRGs) in the THP-1 cells treated with 50 μg/mL (low dose) and 100 μg/mL (high dose) of FeNPs, respectively. Under the same conditions, 221 and 265 genes were identified as FeRGs in the HepG2 cells. More genes were regulated by the high-dose FeNPs (hdFeNPs) in the two cell lines, especially in the THP-1 cells. In the THP-1 cells, 229 genes were induced and 58 genes were repressed by the low-dose FeNPs (ldFeNPs), whereas 571 genes were induced and 143 genes were repressed by the hdFeNPs. In the HepG2 cells, 139 genes were induced and 82 genes were repressed by the ldFeNPs, whereas 96 genes were induced and 169 genes were repressed by the hdFeNPs. More genes were induced in the THP-1 cells but repressed in the HepG2 cells. The previous study revealed that more genes were repressed in the RAW264.7 cells but induced in the Hepa1-6 cells [10]. These data indicate that the FeNPs resulted in differential effects on the gene expression patterns of the cells of two species. The expression of some representative genes was confirmed by quantitative PCR (qPCR) analysis (Figure 2).

**Figure 3:**
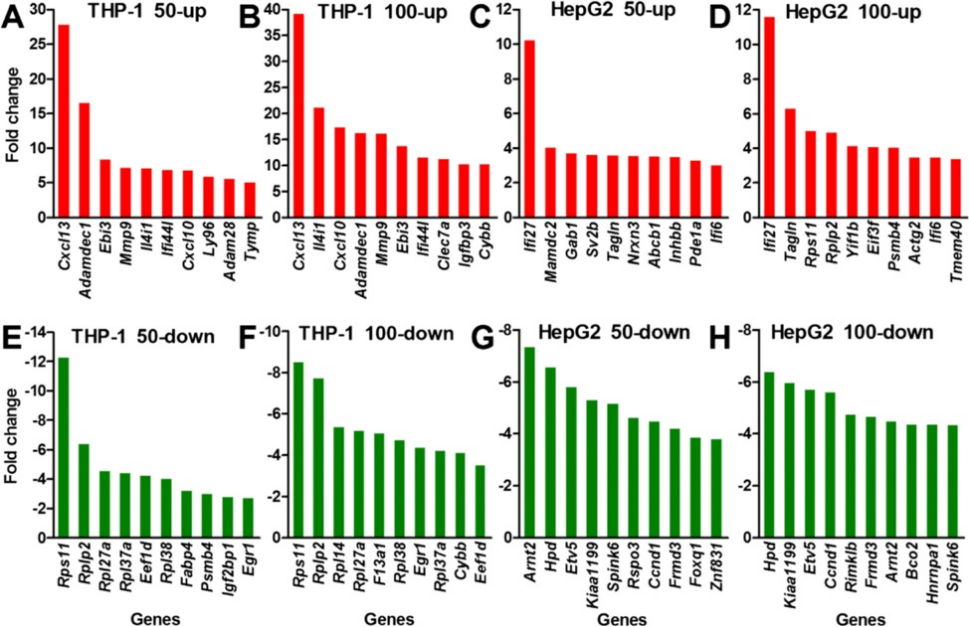
**The top 10 genes with the highest expression changes in the two cell types after treatment with FeNPs. A** and **B**: Induced genes in the THP-1 cells. **C** and **D**: Induced genes in the HepG2 cells. **E** and **F**: Repressed genes in the THP-1 cells. **G** and **H**: Repressed genes in the HepG2 cells. 50-up and 100-up: induced genes in cells treated with 50 μg/mL and 100 μg/mL of FeNPs, respectively; 50-down and 100-down: repressed genes in cells treated with 50 μg/mL and 100 μg/mL of FeNPs, respectively. Some uncharacterized genes with fold changes greater than the lowest fold changes in these plots are not shown in this figure.

The genes with highest fold change in transcription revealed the most sensitive responses of the cells to FeNPs at the gene expression level. The top 10 induced and repressed FeRGs in the two cell lines are shown in Figure 3. Clearly, the top FeRGs in two cell lines are completely different. In the THP-1 cells, *Cxcl13* was the gene most significantly induced by both doses of FeNPs. *Cxcl13* is a strong humoral immune response gene in various neuroinflammatory diseases [27]. Other FeRGs including *Adamdec1*, *Ebi3*, *Ifi44l*, *Clec7a* and *Ly96* are also related to immune responses. *Mmp9* plays a critical role in the positive regulation of the apoptotic process [28]. In the THP-1 cells, most of the most strongly repressed FeRGs encode ribosomal proteins (i.e., *Rps11*, *Rplp2*, *Rpl14*, *Rpl27a*, *Rpl37a* and *Rpl38*). The top FeRGs indicate that the FeNPs resulted in strong activation of defense responses and repression of protein synthesis in the THP-1 cells. Importantly, 7 FeRGs were highly induced, and 7 others were repressed by both ldFeNPs and hdFeNPs in the THP-1 cells (Figure 3), indicating that these effects were stable in the THP-1 cells treated with different doses of FeNPs.

In the HepG2 cells, 3 genes, *Ifi27*, *Ifi6I* and *Tagln*, were highly induced by both the ldFeNPs and hdFeNPs (Figure 3). *Ifi27* was the gene that was most significantly induced by both ldFeNPs and hdFeNPs. Another *Ifi* gene, *Ifi6*, was also significantly induced by both doses of the FeNPs. *Ifi27* and *Ifi6* are associated with immune responses [29,30]. *Ifi27* codes for a mitochondrial protein that contributes to IFN-induced cell death and apoptosis through perturbation of normal mitochondrial function [31]. *Tagln*, which is associated with cell migration, was also induced genes in the HepG2 cells treated with both doses of FeNPs [32]. Seven genes were among the most significantly repressed genes in the HepG2 cells treated with both ldFeNPs and hdFeNPs. *Arnt2* and *Etv5* encode DNA-binding transcription factors (TFs). The TF encoded by *Arnt2* acts as a partner for several sensor proteins that bind the regulatory DNA sequences in genes responsive to developmental and environmental stimuli. The TF coded by *Etv5* is a member of the ETS family. *Hpd* encodes an enzyme in the catabolic pathway of tyrosine; *KIAA1199* (*Cemip*) encodes a cell migration-inducing protein; *Spink6* encodes a serine protease inhibitor selective for kallikreins; and *Frmd3* encodes a putative tumor suppressor protein. *Ccnd1* encodes cyclin D1 of the cyclin family, which functions as regulator of the CDK kinases CDK4 or CDK6, which are required for the cell cycle G1/S transition. Clearly, most of these top repressed genes are involved in cell growth, proliferation and migration.

**Figure 4:**
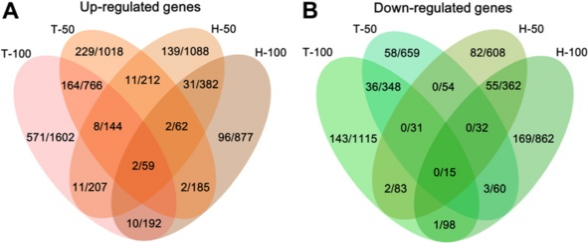
**Comparison of FeRGs in the THP-1 and HepG2 cells. A**: Comparison of induced genes in the two cell lines. **B**: Comparison of repressed genes in the two cell lines. Each Venn diagram is divided into four areas labeled as T-50, T-100, H-50 and H-100. T-50 and T-100, THP-1 treated with 50 μg/mL and 100 μg/mL of FeNPs, respectively. H-50 and H-100, HepG2 cells treated with 50 μg/mL and 100 μg/mL of FeNPs, respectively. The number in overlapped area represents the overlapping genes. The numbers before and after the slash represent the genes with fold changes greater than 2 and 1.5, respectively.

**Figure 5:**
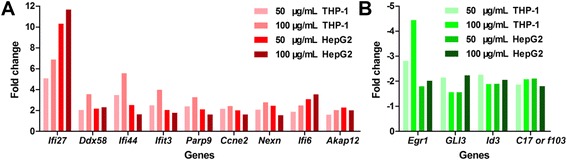
**Commonly regulated genes in the THP-1 and HepG2 cells. A**: The induced genes and their expression levels in the two types of cells treated with two doses of FeNP. **B**: The repressed genes and their expression levels in the two types of cells treated with two doses of FeNP.

The genes commonly regulated in both cell lines revealed the common responses of cells to the FeNPs at the gene expression level. A four-way Venn analysis revealed that 2 genes (*Ifi27* and *Ddx58*) were commonly induced by two doses of FeNPs in these two cell lines (Figure 4A). Eleven genes (*Ifi27*, *Ifi44*, *Ifit3*, *Ddx58*, *Fbxo16*, *Parp9*, *Serpini1*, *Usp25*, *Ccne2*, *Nexn*, and *Rg9mtd2*) were commonly induced by ldFeNPs in both cell lines, and 10 genes (*Ifi27*, *Ifi6*, *Ddx58*, *Akap12*, *Col9a2*, *Nampt*, *Narg1*, *Tmed2*, *Usp16*, and *Zcchc2*) were commonly induced by hdFeNPs in both cell lines (Figure 4A). However, no genes were commonly repressed by both doses of FeNPs in both cell lines (Figure 4B). Only one gene (*Egr1*) was commonly repressed by the hdFeNPs in both cell lines (Figure 4B). Further identification of genes with fold changes greater than 1.5, but at least in one of the cases, greater than 2.0, revealed that 9 and 4 genes were induced and repressed, respectively by both doses of FeNPs in the two cell lines (Figure 5). Among these genes, *Ifi27*, *Ifi44*, *Ifi6* and *Ifit3* express interferon-induced proteins as a defense response to viruses, and *Ddx58* is involved in the viral double-stranded (ds) RNA recognition and the regulation of immune response. These genes indicate that the FeNPs induced cellular responses in the treated cells similar to those induced by viruses [33]. *Parp9* and *Nexn* are associated with cell migration. *Ccne2* belongs to the highly conserved cyclin family and is involved in cell division [34]. *Akap12* encodes a cell proliferation-related protein [35,36]. The repressed genes, *Egr1* and *GLI3*, encode the C_2_H_2_-type zinc-finger proteins of the EGR family, which play roles in cell proliferation [37,38]. *Id3* is associated with cell growth [39,40].

### Cluster analysis of FeNP-responsive genes

**Figure 6:**
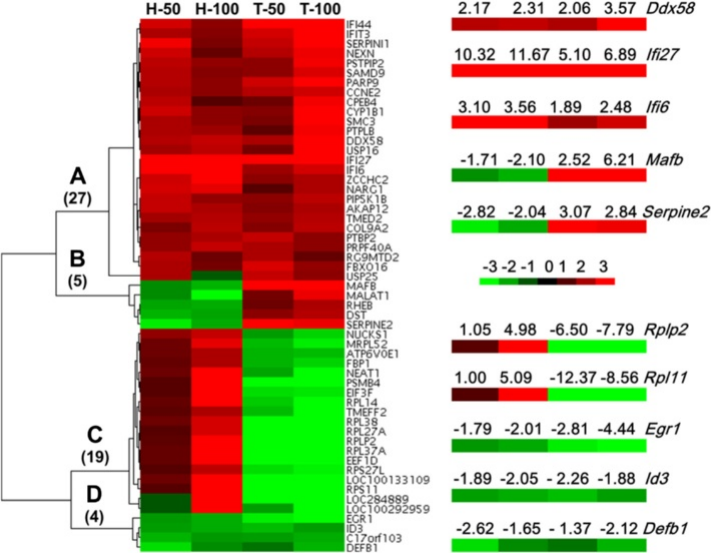
**Cluster analysis of genes. Fifty-five FeRGs from the two cell lines were clustered according to their expression levels using a hierarchical clustering.** The heatmap was drawn with Java TreeView. Red and green represent up- and down-regulation, respectively. The color depth reflects the expression level between −3 and +3 (marker). The numbers of genes in Clusters **A** to **D** are shown in parentheses. The fold changes of 10 representative genes in four clusters are shown in the zoomed images. T-50, H-50, T-100, and H-100, see Figure 4.

The four-way Venn analysis found that only 2 genes were commonly regulated by both doses of FeNPs in the two cell lines (Figure 4). To identify additional commonly regulated genes in these two cell lines, the genes differentially expressed under at least one treatment in each of two cell types were identified. As a result, 55 commonly regulated genes were found (Figure 6). The hierarchical clustering analysis revealed that these genes were classified into four clusters. Clearly, some genes were consistently induced or repressed in both types of cells (Clusters A and D), whereas some genes were inversely regulated in the two cell lines (Clusters B and C). The former reveals the cell-independent effects, whereas the latter reveals the cell-specific effects of the FeNPs on the gene expression in these two cell lines.

### Functional annotation of FeNP-responsive genes

**Figure 7:**
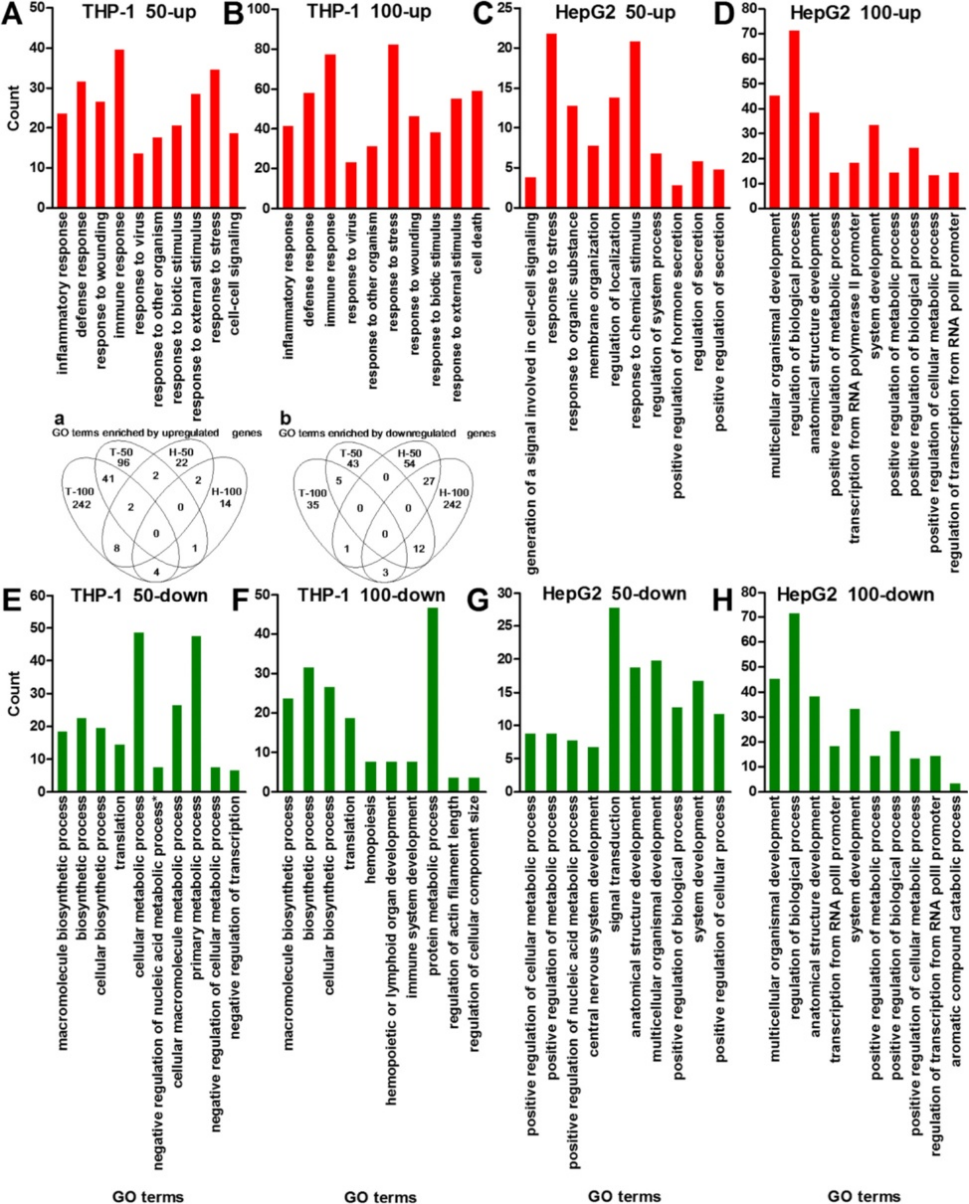
**Top 10 GO terms enriched by induced and repressed genes in the two cell lines. A** and **B**: GO terms enriched by the induced genes in the THP-1 cells. **C** and **D**: GO terms enriched by the induced genes in the HepG2 cells. **E** and **F**: GO terms enriched by the repressed genes in the THP-1 cells. **G** and **H**: GO terms enriched by the repressed genes in the HepG2 cells. *Negative regulation of nucleobase, nucleoside, nucleotide and nucleic acid metabolic process. PolII, polymerase II. The p values for all GO terms are less than 0.05. In each plot, the GO terms were aligned from left to right according to their p values from low to high. 50-up, 100-up, 50-down, and 100-down, see Figure 3. Inset: Venn analysis of all GO terms enriched by the induced genes (a) and repressed genes (b) in the THP-1 and HepG2 cell lines. T-50, H-50, T-100, and H-100, see Figure 4. The numbers in overlapped areas represent the overlapping GO terms.

The GO analysis revealed that the inflammatory, defense and immune responses, and the responses to stress, wounding, external stimuli, biotic stimuli, viruses, and other organisms were most significantly enriched in the THP-1 cells (Figure 7). The inflammatory and defense responses were the two biological processes most significantly enriched among the genes induced by both ldFeNPs and hdFeNPs in the THP-1 cells (Figure 7). In combination with the common enrichment of the responses to viruses in the cells treated with both doses of FeNPs and the similar particulate structure of FeNPs and virus, it seems that the THP-1 cells recognized FeNPs as viruses and responded with virus-like cellular effects. Similar responses were also found in the RAW264.7 cells [10], revealing that the virus-like cellular effects are a common cytotoxic response to the FeNPs in the monocyte-macrophage system. In addition, the hdFeNPs activated many more genes of these response-related biological processes in the THP-1 cells (Figure 7), indicating that the hdFeNPs induced more intense virus-like cellular effects. The exacerbated cytotoxicity induced by the hdFeNPs is also indicated by the activation of the biological process of cell death in cells treated with the hdFeNPs (Figure 7), which agrees with the significant apoptosis of the THP-1 cells that resulted from the treatment with 100 μg/mL of the same nanoparticles [7]. In the HepG2 cells, several response-related biological processes were also enriched among the genes induced by ldFeNPs, similar to those observed in the Hepa1-6 cells treated with the hdFeNPs [10].

**Figure 8:**
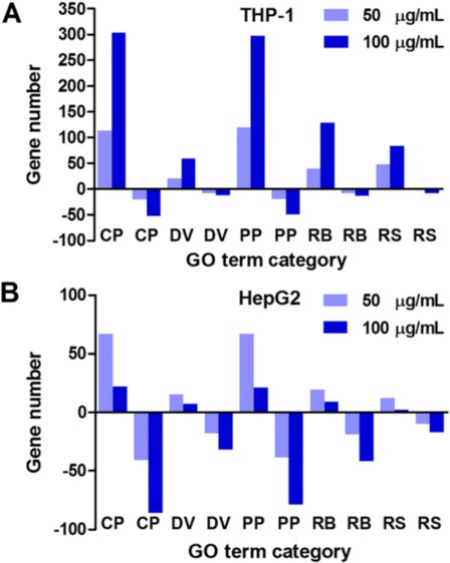
**Distribution of FeRGs in the first-level GO category. A**: FeRGs in the THP-1 cells. **B**: FeRGs in the HepG2 cells. GO terms (p < 0.01) were classified into five groups at the first level of Biological Processes. CP, cellular process; DV, development; PP, physiological process; RB, regulation of biological process; RS, response to stimulus. The bars above and below the abscissa represent the induced and repressed genes, respectively.

To evaluate the distribution of FeRGs among various biological processes, the GO terms were classified into five categories at the first level, and the FeRGs belonging to each category were counted. The results demonstrate that the hdFeNPs significantly increased the induced genes in each category in the THP-1 cells but decreased the induced genes in each category in the HepG2 cells (Figure 8). This is completely different from the effects observed in the mouse cell lines. Specifically the hdFeNPs decreased the numbers of the induced genes in these categories in the RAW264.7 cells but increased the numbers of the induced genes in these categories in the Hepa1-6 cells [10]. However, the hdFeNPs increased the numbers of the repressed genes in each category in both two human (Figure 8) and mouse cell lines [10]. These data reveal that two human cell lines responded to the two doses of FeNPs with the different patterns of gene expression. These data also demonstrate that the FeNPs induced different forms of nanotoxicity in cell types with different biological functions.

**Figure 9:**
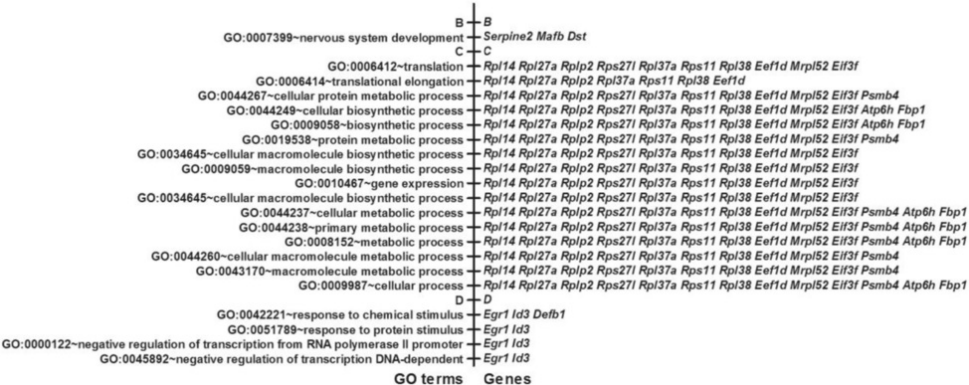
**GO analysis of the FeRGs with different expression patterns.** B, C and D, genes in Clusters B, C and D (Figure 6), and their associated GO terms. The p values for all GO terms are less than 0.05. An alias of *Atp6h* is *Atp6v0e1*.

To identify the biological processes associated with the genes that were consistently regulated by two doses of FeNPs in two cell lines (clustered in Figure 6), a new GO analysis was performed with these genes. The results revealed that 4 biological processes were enriched mainly by two commonly repressed genes (*Egr1* and *Id3*), whereas 16 biological processes were enriched by 13 genes that were inversely regulated in the two cell lines (Figure 9). The 16 biological processes were all clearly associated with protein translation and were primarily determined by 7 nucleosomal proteins (*Rpl14, Rpl27a, Rplp2, Rpl37a, Rps11, Rpl38*, and *Rps27l*), a translation initiation factor (*Eif3f*) and a translation elongation factor (*Eef1d*). These genes were induced in the HepG2 cells but repressed in the THP-1 cells, indicating that the FeNPs significantly induced protein production in the HepG2 cells but repressed this process in the THP-1 cells. These results are consistent with the results of our recent evaluation of cell viability, which indicated that the ldFeNPs significantly (p < 0.05) and hdFeNPs very significantly (p < 0.01) decreased the viability of the THP-1 cells, but neither the ldFeNPs nor the hdFeNPs affected the viability of the HepG2 cells (not shown). Interestingly, a gene encoding a mitochondrial ribosomal protein L52 (*Mrpl52*) was also significantly repressed in the THP-1 cells but induced in the HepG2 cells by the FeNPs (Figure 9), indicating that both cytoplasmic and mitochondrial protein production were significantly affected by the FeNPs. In addition, one biological process (nervous system development) was represented by 3 genes repressed in HepG2 but induced in THP-1 (*Serpine2*, *Mafb* and *Dst*). Such inverse regulation of a biological process demonstrates the typical cell-specific effects of FeNPs.

### Pathway analysis of FeNP-responsive genes

**Figure 10:**
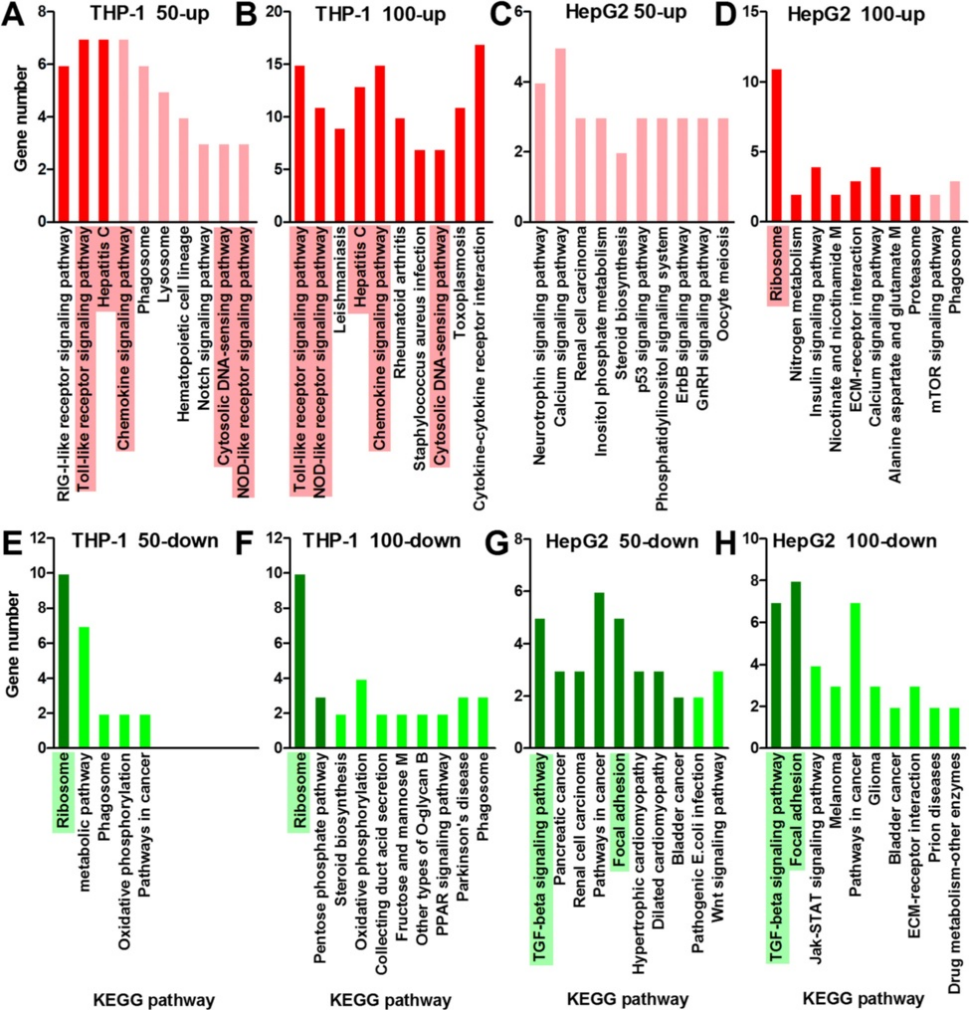
**KEGG pathways enriched as indicated by the FeRGs. A** and **B**: KEGG pathways enriched by the induced genes in the THP-1 cells. **C** and **D**: KEGG pathways enriched by the induced genes in the HepG2 cells. **E** and **F**: KEGG pathways enriched by the repressed genes in the THP-1 cells. **G** and **H**: KEGG pathways enriched by the repressed genes in the HepG2 cells. The pathways with p values less than 0.05 are shown as bars in deep colors. The pathways with p values over 0.05 are shown as bars in light colors. In each plot, the pathways were aligned from left to right according to their p values from low to high. Abbreviations in the pathway names: M, metabolism; B, biosynthesis. The pathway that was significantly activated by both doses of FeNPs is highlighted by shading.

The most significantly (p < 0.05) enriched KEGG pathways are shown in Figure 10. In the THP-1 cells, the Toll-like receptor (TLR) signaling pathway is significantly enriched by the FeRGs that were induced by both ldFeNPs and hdFeNPs. TLRs are membrane-bound receptors expressed on innate immune cells such as macrophages and dendritic cells, which generate innate immune responses [41,42]. The TLR signaling pathway was reported to be activated by the FeNPs in RAW264.7 cells [9], and by ceramic (TiO_2_, SiO_2_ and ZrO_2_) and metallic (cobalt) nanoparticles in a human myelomonocytic cell line (U-937) [41]. In addition, the RIG-I-like receptor and chemokine signaling pathways were significantly enriched by the FeRGs induced by both ldFeNPs and hdFeNPs. The cytosolic DNA-sensing pathway and the NOD-like receptor signaling pathway were significantly enriched by the FeRGs induced by hdFeNPs. All these pathways are known to play critical roles in immunological responses [43,44]. Additionally, more genes in these pathways were induced by hdFeNPs. These data indicate that the hdFeNPs induced more intense immunological responses than the ldFeNPs in the THP-1 cells. This is consistent with the results of the GO analysis that revealed that the biological process of immune response was consistently highly enriched by the FeRGs induced by both ldFeNPs and hdFeNPs; however, the ldFeNPs induced 40 immune response-related FeRGs, but the hdFeNPs induced 78 immune response-related FeRGs (Figure 7). The more intense reactions of the cells to the hdFeNPs were also demonstrated by the significant activation of five human disease pathways by hdFeNPs, including the Leishmaniasis, Rheumatoid arthritis, Staphylococcus aureus infection, Toxoplasmosis, and Malaria pathways (Figure 10).

**Figure 11:**
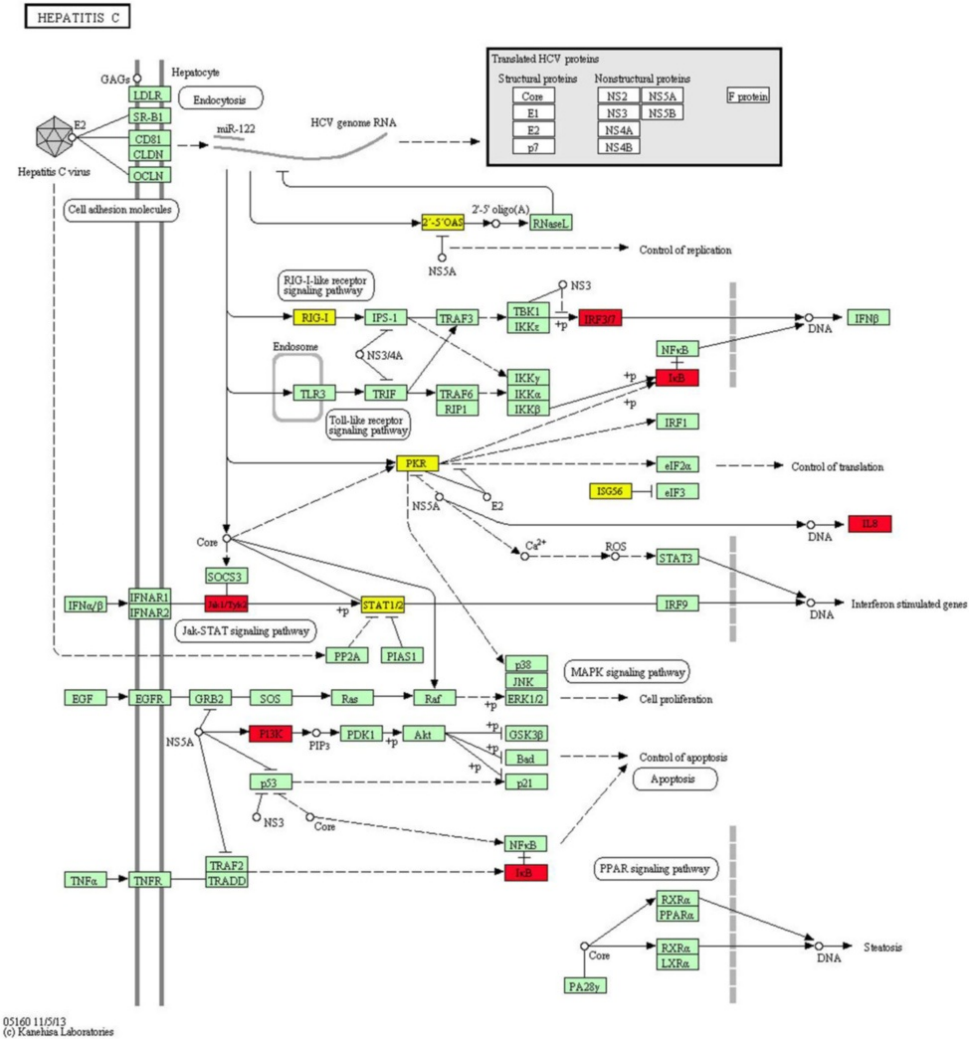
**KEGG pathway of hepatitis C in the FeNP-treated THP-1 cells.** The genes in red refer to the FeRGs induced by 100 μg/mL of FeNPs. The genes in yellow refer to the FeRGs induced by both 50 μg/mL and 100 μg/mL of FeNPs. Abbreviations for the KEGG parameters can be found on the KEGG pathway webpage.

It is interesting that the hepatitis C pathway is significantly enriched by the FeRGs induced by both the ldFeNPs and hdFeNPs in the THP-1 cells (Figure 10). Moreover, more genes in this pathway were induced by the hdFeNPs (Figures 10 and 11). In this pathway, the gene *Oas1*, *Oas2* and *Oas3* were induced by both doses of FeNPs (Figure 11). These genes encode the 2′,5′-oligoadenylate (2-5A) synthetases, enzymes that play essential roles in the innate immune response to viral infection [45,46]. In this pathway, the gene *Ddx58* (coding RIG-I) was also induced by both ldFeNPs and hdFeNPs (Figure 11). RIG-I functions as a pattern recognition receptor that is a sensor for viruses such as hepatitis C virus. The activation of RIG-I-like receptor signaling pathway can induce the production of interferon [47], which is supported by the significant overexpression of many interferon-related genes, including *Ifi27*, *Ifi6*, *Ifi16*, *Ifi35*, *Ifi44*, *Ifi44l*, *Ifit1*, *Ifit2*, *Ifit3*, *Ifit5*, *Ifitm1*, *Ifih1*, *Isg20*, and *Irf7*. The GO analysis also revealed that the biological process of response to viruses was significantly enriched by the FeRGs induced by the two doses of FeNPs in the THP-1 cells (Figure 7). These data indicate that the FeNPs act on the THP-1 cells similarly to the responses to viruses including the hepatitis C virus. This is also in consistent with the fact the hydrodynamic size of the FeNPs (41 nm) (Figure 1) used in this study is similar to the size of the hepatitis B virus particles (42 nm).

In the THP-1 cells, only the ribosomal pathway was significantly enriched by the FeRGs repressed by both ldFeNPs and hdFeNPs (Figure 10E and F). In this pathway, 10 genes coding for ribosome proteins, including *Rps27l*, *Rplp2*, *Rps11*, *Rps19*, *Rpl14*, *Rpl27*, *Rps10*, *Rpl38*, *Rpl37a*, and *Rpl27a*, were commonly repressed by both doses of FeNPs. The extensive repression of these genes suggests that the FeNPs significantly inhibited protein production in this cell. The similar repression of ribosomal pathway was also found in the zebra fish embryos exposed to silver nanoparticles [22]. However, the ribosomal pathway was also the most significantly enriched by the FeRGs induced by the hdFeNPs in the HepG2 cells (Figure 10D). In this cell type, 11 genes coding ribosome proteins, including *Rps27l*, *Rplp2*, *Rps11*, *Rps19*, *Rpl14*, *Rpl27*, *Rps10*, *Rpl38*, *Rpl37a*, *Rpl27a*, and *Rps6*, were significantly induced by hdFeNPs. Except for *Rps6*, these genes are identical to those altered in the THP-1 cells. These data indicate that the ribosomal pathway was inversely regulated by the FeNPs in two cell lines due to the opposite regulation of a same set of genes coding ribosome proteins. Such differential regulation of a set of genes and their involved pathway demonstrate a typical form of cell-specific toxicology of a nanomaterial.

In the HepG2 cells, no pathway was significantly enriched by the genes induced by the ldFeNPs (Figure 10C). However, 8 pathways were significantly enriched by the genes induced by the hdFeNPs (Figure 10D). These pathways are mainly associated with cellular metabolism. The ribosome pathway was highly enriched by the FeRGs induced by the hdFeNPs. Proteasomes are responsible for protein metabolism (proteolysis). Three pathways are responsible for the metabolism of other nitrogen-containing materials, including the nitrogen, nicotinate and nicotinamide, and alanine, aspartate and glutamate pathways. The insulin signaling pathway is responsible for glucose metabolism. In addition to these metabolism-related pathways, the ECM-receptor interaction and calcium signaling pathways were also significantly enriched by the genes induced by hdFeNPs in HepG2. Two extracellular matrix (ECM) macromolecules, vitronectin (encoded by *Vtn*) and collagen (encoded by *Col6a1*), which benefit cell proliferation and migration, were significantly induced by the hdFeNPs. Four genes (*P2rx4*, *Tnnc1*, *Pde1a*, and *Erbb4*) in the calcium signaling pathway, which is closely associated with cell adhesion, were significantly induced by hdFeNPs. Therefore, the pathways enriched by the induced genes in the HepG2 cells were mainly associated with cellular metabolism, proliferation, migration and adhesion.

In the HepG2 cells, two pathways, TGF-beta signaling and focal adhesion, were significantly enriched by the FeRGs repressed by both the ldFeNPs and hdFeNPs (Figure 10G and H). Furthermore, more genes in these two pathways are repressed by the hdFeNPs, indicating that the hdFeNPs exert greater effect on the HepG2 cells than the ldFeNPs. This dose-dependent effect can be observed in the THP-1 cells (Figure 10A and B). Importantly, the TGF-beta signaling pathway was most significantly enriched by the genes repressed by both doses of the FeNPs in the HepG2 cells. This pathway is the most important pathway responsible for cell viability. Furthermore, a wide spectrum of cellular functions such as proliferation, apoptosis, differentiation and migration are regulated by TGF-β family members [48]. In the HepG2 cells, 5 (including *Id1*, *Id2*, *Bmp6*, *Smad9*, and *Tgfb1*) and 7 (including *Id1*, *Id2*, *Id3*, *Bmp6*, *Smad6*, *Smad7*, and *Smad9*) genes in this pathway were repressed by the ldFeNPs and hdFeNPs, respectively. *Smad6*, *Smad7* and *Smad9* encode proteins of the SMAD family that act as signal transducers and transcriptional modulators [49]. *Id1*, *Id2* and *Id3* encode helix-loop-helix (HLH) proteins that function as dominant-negative regulators of basic HLH (bHLH) transcription factors by forming inactive heterodimers with intact bHLH. ID proteins play important roles in control of cell growth, differentiation and tumorigenesis [50,51]. The down-regulation of these genes can promote cell growth and increase the risk of tumorigenesis [52]. These data suggest that the FeNPs may stimulate the growth of the HepG2 cells. This is supported by the results of cell viability assay, which revealed that neither the ldFeNPs nor the hdFeNPs affected the viability of the HepG2 cells, and the hdFeNPs even enhanced cell viability (106.4% of the untreated control cells) (not shown). This is also in agreement with the activation of the ribosomal pathway and several metabolism-related pathways by the hdFeNPs in this cell.

The focal adhesion pathway was also significantly enriched by the FeRGs repressed by both the ldFeNPs and hdFeNPs in the HepG2 cells. The pathway plays an essential role in important biological processes including cell motility, cell proliferation, cell differentiation, regulation of gene expression, and cell survival [53]. In the HepG2 cells, 4 (*Ccnd1*, *Fyn*, *Fn1*, *Itgb5*, and *Vegfc*) and 8 (*Ccnd1*, *Fyn*, *Fn1*, *Itgb5*, *Col3a1*, *Vav3*, *Igf1r*, and *Egfr*) genes of this pathway were significantly repressed by the ldFeNPs and hdFeNPs, respectively. The down-regulation of these important cell adhesion-related genes suggests that the growth and mobility of the cells were enhanced by the FeNPs, which is consistent with the slight increase of cell viability and activation of ribosomal pathway. The enhancement of cell growth and mobility may also increase the risk of tumorigenesis. This is supported by the enrichment of the TGF-beta signaling pathway and several cancer-related pathways including those associated with pancreatic cancer, renal cell carcinoma, and bladder cancer in the HepG2 cells (Figure 10G). In the HepG2 cells, these cancer-related pathways are mainly enriched by the genes *Ccnd1*, *Vegfc*, *Tgfb1*, *Arnt2*, *Fn1*, and *Fzd10*.

In the pathway analysis, it was found that the phagosome pathway was also enriched by several genes in both the THP-1 and HepG2 cells. In the THP-1 cells, this pathway was enriched by 6 genes induced by ldFeNPs (*Atp6v1h*, *Hla-Dra*, *Clec7a*, *Cd14*, *Cd36*, and *Cybb*), 2 genes repressed by ldFeNPs (*Calr* and *Atp6v0e1*), and 3 genes repressed by hdFeNPs (*Calr*, *Atp6v0e1*, *Atp6v1c2*) (Figure 9D and E). In the HepG2 cells, this pathway was enriched by 3 genes induced by hdFeNPs (*Calr*, *Atp6v0e1*, and *Ctss*) (Figure 10F). Clearly, the genes *Calr* and *Atp6v0e1* were commonly regulated by the FeNPs in both cell lines; however, the expression of these genes was inversely regulated in the two cell lines. This indicates once again the inverse toxicity of the FeNPs in cells of different functions. However, these genes demonstrated the endocytotic activity of two cells to the FeNPs, which agrees with the previous reports that the FeNPs were internalized into cells by endocytosis [54-57]. In addition to the phagosome pathway, the lysosome and notch signaling pathways were also enriched by the genes induced by ldFeNPs in the THP-1 cells (Figure 10A). In this cell line, the lysosomal pathway was enriched by 5 genes (*Atp6v1h*, *Laptm4b*, *Ap1s3*, *Ctsh*, and *Lamp3*), and the notch signaling pathway was enriched by 3 genes (*Maml2*, *Lfng*, and *Dtx4*) (Figure 10A). These results agree with many previous reports that FeNPs are accumulated into the lysosomes [13,42].

### Comparison of human cells with mouse cells

**Figure 12:**
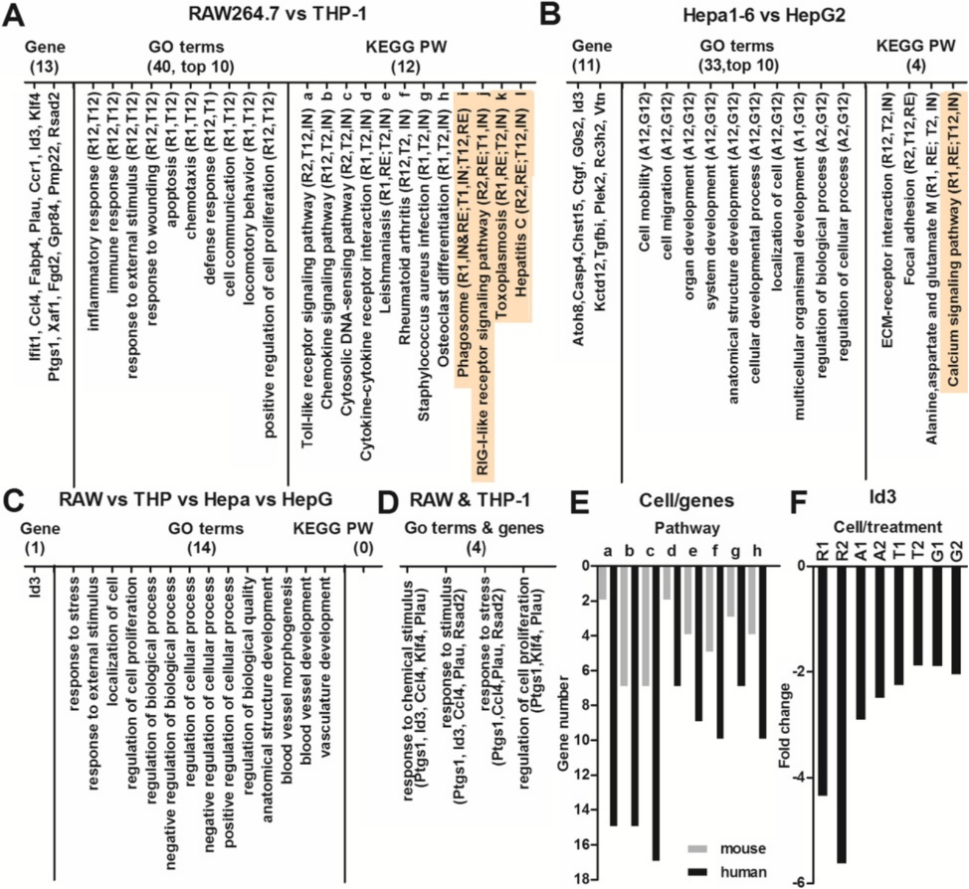
**Comparison of the FeRGs, GO terms and KEGG pathways for all four cell types. A**: Common genes, GO terms and KEGG pathways for the RAW264.7 and THP-1 cells. **B**: Common genes, GO terms and KEGG pathways for the Hepa1-6 and HepG2 cells. **C**: Common genes, GO terms and KEGG pathways for all four cell lines. RAW, RAW264.6; THP, THP-1; Hepa, Hepa1-6; HepG, HepG2. All GO terms and KEGG pathways have p values < 0.05 except for five KEGG pathways with p value > 0.05 (indicated by shading). **D**: GO analysis of genes common to the RAW264.7 and THP-1 cells. **E**: Comparison of the numbers of FeRGs in 8 pathways of the THP-1 and RAW264.7 cells. a to h, pathways in image A. **F**: Expression of *Id3* gene in the four cell lines treated with FeNPs. Symbols of cells and treatments in image A, B and F: R, RAW264.6; T, THP-1; A, Hepa1-6; G, HepG2; 1, treated with 50 μg/mL of FeNPs; 2, treated with 100 μg/mL of FeNPs. For example, T1 and T2 indicate THP-1 cells treated with 50 μg/mL and 100 μg/mL of FeNPs, respectively. R12, R1 and R2; T12, T1 and T2; A12, A1 and A2; G12, G1 and G2. IN, enriched by induced genes; RE, enriched by repressed genes. M, metabolism.

The effects of the same FeNPs at the same two doses on the gene expression profiles of two mouse cell lines (RAW264.7 and Hepa1-6) were recently investigated by our lab [10]. To identify the effects of the FeNPs on the cells of different species, this study used the human equivalents of mouse cells. THP-1 and RAW264.7 are cell lines of monocyte-macrophage system, whereas HepG2 and Hepa1-6 are hepatoma cell lines. The genes with changes ≥1.5-fold, the GO terms, and the KEGG pathways for the four cell types were systematically compared. The genes with one or two changes ≥ 2.0-fold among the compared cells were identified as the common genes. The common genes, GO terms, and KEGG pathways identified in the two monocyte-macrophage cell lines, the two hepatoma cell lines, and all four cell lines were shown in Figure 12.

Although some pathways were significantly (p < 0.05) enriched in a manner common to both human and mouse cells, the FeRGs involved in these pathways were quite different. For example, in 3 commonly enriched pathways of the two liver cells (Figure 12B), only the focal adhesion pathway shared a gene (*Col3a1*) between the human and mouse cells. In the 8 pathways enriched in common to the two types of blood cells (Figure 12A), only two genes (*Ccl4* and *Ccl5*) were shared by the human and mouse cell lines. The *Ccl4* and *Ccl5* genes shared by two cell lines are involved in the TLR signaling pathway, chemokine signaling pathway, and cytosolic DNA-sensing pathway. The gene *Ccl4*, shared by two cells, is in the cytokine-cytokine receptor interaction pathway. The gene *Ccl5* shared by two cells is in the rheumatoid arthritis pathway. Additionally, it was found that the most common pathways were enriched by the induced or repressed genes in the cells of both species. For example, the TLR signaling pathway is enriched by the induced genes in both human and mouse cells, but the focal adhesion is enriched by the repressed genes in both human and mouse cells (Figure 12A and B). However, two other pathways were inversely regulated in cells of two species. Specifically, the alanine, aspartate and glutamate metabolism and Leishmaniasis pathways were enriched by repressed genes in mouse cells but the induced genes in human cells (Figure 12A and B).

The GO analyses of the common genes revealed that four biological processes were significantly (p < 0.05) enriched in the two blood cell types. These biological processes are mainly related to the responses of the cells to stimuli and stress (Figure 12D). The GO analyses of the common genes revealed that no biological processes were significantly (p < 0.05) enriched in the two liver cells. However, the biological processes of cell adhesion and cell-matrix adhesion were enriched by three genes, *Ctgf*, *Tgfbi* and *Vtn*, in the two liver cells. It seems that the THP-1 cells are more sensitive to the FeNPs than the RAW264.7 cells because in the 8 common pathways (Figure 12A), many more genes were induced by the FeNPs in the THP-1 cells (Figure 12E). For instance, in the TLR signaling pathway, only two genes (*Ccl4* and *Ccl5*) were significantly induced by the FeNPs in the RAW264.7 cells, however, as many as 15 genes (*Ccl4*, *Ccl5*, *Cxcl10*, *Cxcl11*, *Cd14*, *Cd40*, *Cd86*, *Il8*, *Il1b*, *Nfkbia*, *Stat1*, *Irf7*, *Ly96*, *Tlr8*, and *Pik3r1*) were significantly induced by the FeNPs in the THP-1 cells (Figure 12E).

Identification of the common genes revealed that only the *Id3* gene was commonly regulated by the FeNP in all four cell lines (Figure 12F). *Id3* (inhibitor of DNA binding 3) was the second most significantly repressed gene in the RAW264.7 cells [10]. Previous studies have demonstrated that ID3 is a redox-sensitive signaling molecule [39,40,58]. *Id3* and *Gklf* were identified as two differentially regulated redox-sensitive genes in vascular smooth muscle cells (VSMC) [40]. *Id3* was induced by xanthine/xanthine oxidase (X/XO) but repressed by Fe^3+^NTA (H-Fe). Conversely, *Gklf* was repressed by X/XO but induced by H-Fe [40]. It was found that *Gklf* could reduce the *Id3* expression by binding to its promoter [40]. It is well known that FeNPs have redox activity and could result in oxidative stress in cells [59-63]. Therefore, the redox-sensitive down-regulation of *Id3* in VSMC is perfectly in agreement with the significant down-regulation in common among the four cell lines treated with two doses of FeNPs. These data suggest that the FeNPs used in this study induced the common down-regulation of *Id3* gene in the four cell lines by its redox activity in cells. These data also show that the down-regulation of the *Id3* gene is a general and sensitive biomarker of the nanotoxicity of the FeNPs, which demonstrates that the redox activity of FeNPs and the resulted oxidative stress are the most significant and prevalent form of the cellular toxicity of the FeNPs. In addition, because *Id3* codes for the ID3 protein of the HLH transcription factor family, which inhibits transcription by forming nonfunctional dimers with other bHLH transcription factors, its significant down-regulation by FeNP suggests that FeNP may disturb the normal biological processes, including cell growth [39], cell differentiation [51], cell apoptosis [64,65], and tumorigenesis [66-68] that are controlled by this transcription factor.

## Conclusion

This study investigated the effects of an 11-nm DMSA-coated magnetite FeNP on the gene expression profiles of two human cell lines, THP-1 and HepG2. It was found that the expression of hundreds of genes was significantly changed by a 24-h treatment of the FeNPs at two doses, 50 μg/mL and 100 μg/mL in the two types of cells. By identifying the FeRGs and annotating their functions, this study characterized the general and cell-specific effects of the FeNPs on two cells at the gene, biological process and KEGG pathway levels. At the doses used, the overall effects of the FeNPs in the THP-1 cells was the induction of various responses and repression of protein translation, but in the HepG2 cells these particles promoted cell metabolism, growth and mobility. This study also characterized the common genes, biological processes and pathways effected by the FeNPs in two human and two mouse cell lines, and identified *Id3* as a nanotoxicity biomarker of FeNPs.

## Methods

### Reagents and cells

The DMSA-coated magnetite FeNPs were supplied by the Gu’s lab of Southeast University (Nanjing, China) [69]. HEPES and glutaraldehyde were purchased from Sigma Aldrich (St. Louis, MO, USA). The Trizol reagent and the DMEM cell culture medium were purchased from Invitrogen Gibco (Carlsbad, CA, USA). The Human Genome U133 Plus 2.0 GeneChips® microarrays were purchased from Affymetrix (Santa Clara, CA, USA). The Reverse Transcriptase Kit was purchased from TaKaRa (Dalian, China). The Fast SYBR Master Mix was purchased from Applied Biosystem (Grand Island, NY, USA). The THP-1 and HepG2 cells were purchased from the China Center for Type Culture Collection (Shanghai, China).

### Characterization of nanoparticles

The DMSA-coated magnetite FeNPs were synthesized by thermal decomposition [69]. The size and dispersibility of the FeNPs were evaluated using a transmission electron microscope (JEM-2100). The hydrodynamic size distribution of the FeNPs was analyzed with a submicron particle analyzer (Beckman Coulter N4 Plus). The surface charge of the FeNPs was measured with a zeta potential analyzer (Beckman Coulter Delsa 440SX).

### Exposure of cells to nanoparticles

The suspension of the FeNPs in water was sterilized by filtration through a 0.22-μm membrane. The human cells were cultured in the DMEM cell culture medium supplemented with 10% fetal calf serum, 100 units/mL penicillin, 100 μg/mL streptomycin and 10 mM HEPES in a humidified 5% CO_2_ atmosphere at 37°C. To treat the cells with FeNPs, the cells were seeded in plates or flasks and cultivated overnight, and the culture medium was then replaced with fresh medium containing one of two concentrations (50 and 100 μg/mL) of FeNPs. The cells were cultivated for 24 h longer. To detect the cellular internalization of FeNPs, the cells were stained with Prussian blue as previously described [7].

### Detection of gene expression with GeneChip microarray

Total RNA was extracted from the FeNP-treated and untreated control cells with Trizol reagent. The total RNA was quantified using a UV spectrophotometer (NanoDrop 1000) and stored at −80°C for later use. To profile the global gene expression, the RNA samples were analyzed using Affymetrix Human Genome U133 Plus 2.0 GeneChips® microarrays according to standard Affymetrix protocols. Briefly, biotin-labeled cDNA was generated from the RNA using an in vitro transcription reaction in the presence of biotin-labeled ribonucleotides. The labeled cDNA was fragmented and hybridized with the Affymetrix Human Genome U133 Plus 2.0 GeneChips® microarrays at 45°C for 16 h in an Affymetrix hybridization oven. After hybridization, the arrays were washed, stained with streptavidin-phycoerythrin, and scanned with the Affymetrix GeneChip Scanner3000 7G. Image quantitation was performed using GeneChip® Operating Software.

### Data analysis of the GeneChip microarray

Following normalization and background filtration, the signal intensity data of the treated cells were compared with those of the control cells. The transcripts with intensity ratios ≥ 2 or ≤ −2 were identified as DEGs [70]. The functional annotation analysis was performed by uploading on the DEGs to DAVID (v6.7 [71]). The enriched GO functions including the biological processes, cellular components and molecular function with p values less than 0.05 were considered significant. The enriched KEGG pathways were determined with DAVID.

### Detection of gene expression with qPCR

One μg of total RNA was reverse transcribed into cDNA by using the Reverse Transcriptase Kit. The cDNA was used as template to quantify the transcription of genes with quantitative PCR on a StepOne Plus instrument (Applied Biosystem) using Fast SYBR Master Mix. The primers were as follows (5′ to 3′): *Adamdec1*: AGA CTG TGA TTG TGG CTC TCC T, TTG TCC TGG CAA GGT AGC ATC T; *Ebi3*: GCT CAG GAC CTC ACA GAC TAC G, GCA GCA GCA AAG CAA GGA CTC; *Cybb*: AGG GTC AAG AAC AGG CTA AGG A, AGC AGG ACT AGA TGA GCC AGA G; *Rps11*: ACA TTC AGA CTG AGC GTG CC, GGA GCT TCT CCT TGC CAG TT; *Id3*: CAC CTT CCC ATC CAG ACA GCC, GCT TCC GGC AGG AGA GGT TC; *Etv5*: CCT GAG AGA CTG GAA GGC AAA, TCA TAG TTC ATG GCT GGC CG; *Tmem40*: ACC GTA TCC ACA GCG TCC TC, ATT GGT CTG GCT TGG TCT CCT; *Hpd*: GCC TCT AGT CCC AGT AGG AG, TCG CCC ATC TCT TTG TTC CA; *Ifi27*: TCC TTC TTT GGG TCT GGC TGA A, CAT GGG CAC AGC CAC AAC TC; *Gapdh*: ATT TGG TCG TAT TGG GCG, CTC GCT CCT GGA AGA TGG. A melting curve analysis was performed at the end of the PCR programs to confirm the specificity of PCR amplification. The relative quantification (RQ) was calculated using the 2^-ΔΔCt^ method.

## Abbreviations

FeNP: Iron nanoparticle
DEG: Differentially expressed gene
TEM: Transmission electron microscopy
DMEM: Dulbecco’s modified Eagle medium
DMSA: m-2,3-dimercaptosuccinic acid
qPCR: Quantitative PCR
ROS: Reactive oxygen species
CNS: Central nervous system
ldFeNPs: Low-dose FeNPs
hdFeNPs: High-dose FeNPs
MMP: Matrix metalloproteinase
FeRGs: FeNP-responsive genes
DAVID: Database for Annotation, Visualization and Integrated Discovery
GO: Gene ontology
KEGG: Kyoto Encyclopedia of Genes and Genomes
HEPES: 4-(2-Hydroxyethyl)-1-piperazineethanesulfonic acid
